# Isolation and Characterization of Carbonosomes from *Pseudomonas* sp. phDV1 Grown Using Phenol as Carbon Source

**DOI:** 10.3390/microorganisms13020369

**Published:** 2025-02-08

**Authors:** Ermis Dionysios Geladas, Alexandros Lyratzakis, Athina Drakonaki, Georgios Gkikas, Georgios Tsiotis

**Affiliations:** Department of Chemistry, University of Crete, 70013 Voutes, Greece; ermisgeladas@gmail.com (E.D.G.); alexlamp2008@gmail.com (A.L.); athinadrakonaki_@hotmail.com (A.D.); chem2861@edu.chemistry.uoc.gr (G.G.)

**Keywords:** *Pseudomonas* sp. strain phDV1, PHB granules, phenol degradation, PHB-associated proteins

## Abstract

The *Pseudomonas* sp. strain phDV1 was found to utilize monocyclic aromatic compounds as a sole carbon source and has a variety of potential applications in the bioremediation and biosynthesis of biodegradable plastics. It was possible to produce polyhydroxybutyrate when cultivated in the presence of monocyclic aromatic compounds as the sole carbon source. This study provides the small-scale optimization for phenol bioremediation and polyhydroxybutyrate production. The bacterium was cultivated in minimal medium supplemented with different concentrations of phenol. The formation and localization of the polyhydroxybutyrate granules (carbonosomes) in the cell were determined after 72 h of cultivation using Nile Red stain in combination with fluorescence microscopy. Analytical HPLC was also used to quantify the PHB content in the cells and to optimize the production. Finally, comparative proteomic analysis of isolated carbonosomes was used to characterize of their protein composition.

## 1. Introduction

The proliferation of petroleum-derived plastics has highlighted the urgent need for sustainable alternatives, with polyhydroxyalkanoates (PHAs) emerging as a promising solution due to their biodegradable nature. PHAs are a type of biopolymer that accumulate in bacteria and are used as a form of energy storage when nutrient limitation occurs [1]. The most studied PHA, and the first discovered by Lemoigne in 1926, is poly(R)-3-hydroxybutyric acid (P(3HB)), commonly referred to as PHB, which is organized intracellularly in the form of granules called carbonosomes [2,3]. Despite its potential, high production costs remain a significant barrier [4]. Therefore, new low-cost substrates, such as toxic wastes, are being tested to reduce these costs. One important category of toxic waste is phenols. Phenols represent a significant threat due to their high toxicity, posing significant risks even at low concentrations [5].

Several bacteria are able to thrive in toxic environments and utilize aromatic organic compounds. *Pseudomonas* sp. phDV1 is an aerobic, Gram-negative bacterium belonging to the *Pseudomonas pseudoalcaligenes* species, capable of degrading aromatic hydrocarbons, namely phenol, toluene, cresols, naphthalene, and 1,2,3-trimethylbenzene [6,7,8]. It produces P(3HB) under stress conditions, utilizing phenol as a carbon source via the meta-cleavage pathway to yield acetyl-coenzyme A, which is a precursor molecule for the production of P(3HB) [6,9].

*Pseudomonas* sp. phDV1 was found to produce enzymes necessary for the synthesis of PHAs under conditions of intense stress and excess carbon sources [6,9]. The production of PHAs involves complex molecular processes orchestrated by a variety of proteins, each of which plays a crucial role in the biosynthesis and regulation of PHA. Among these proteins, PHA synthase (PhaC) stands out as the key enzyme responsible for catalyzing the conversion of (R)-3-hydroxyacyl-CoA thioester substrates into PHA, thereby initiating the formation of PHA granules [10]. PHA synthase is essential for PHA granule formation, underlining its central role in the biosynthetic pathway. Conversely, PHA depolymerase (PhaZ) facilitates the degradation of PHAs, allowing their utilization as an energy source [10,11].

Complementing the enzymatic machinery involved in PHA metabolism, phasins play a crucial dual role in preventing aggregate formation and inhibiting non-specific binding to PHA granules [11,12]. Although not essential for PHA accumulation, the composition and abundance of phasins correlate strongly with PHA, highlighting their regulatory importance [13]. In addition, regulatory proteins such as PhaR exert critical control over PHA synthesis and regulation in bacteria. As a repressor protein, PhaR binds to the PhaP promoter, modulates its expression and regulates the synthesis of PHB granules [14,15].

The production process of PHB takes place in three sequential steps, each catalyzed by specific enzymes. The process is initiated by the acetyltransferase acetyl-CoA or β-ketothiolase (PhaA), which facilitates the formation of acetoacetyl-CoA from acetyl-CoA molecules. Next, acetoacetyl-CoA reductase (PhaB) converts acetoacetyl-CoA into (R)-3-hydroxybutyryl-CoA, further promoting PHA biosynthesis. Finally, PHA synthase (PhaC) orchestrates the synthesis of ester bonds, which are critical for PHA polymerization [11,16]. Analysis of the complete genome of *Pseudomonas* sp. phDV1 revealed a cluster of genes controlling PHA production and storage, including genes encoding enzymes involved in PHA synthesis (PhaC), PHA degradation (PhaZ), and a putative transcription factor (PhaR) [9].

In this study, we aim to optimize phenol bioremediation and P(3HB) production by the *Pseudomonas* sp. phDV1 strain using phenol as the sole carbon source. This study focuses on small-scale cultivation experiments supplemented with different concentrations of phenol, assessment of P(3HB) granule formation, quantification of PHB content, and characterization of the protein composition of isolated carbonosomes.

## 2. Materials and Methods

### 2.1. Cultivation of Pseudomonas sp. phDV1

*Pseudomonas* sp. strain phDV1 was isolated from a petroleum-contaminated site in Denmark and characterized using whole-genome analysis and full-gene 16rDNA sequence analysis [7,9]. The complete genome sequence was submitted to GenBank and is available under the accession number CP031606 [9]. *Pseudomonas* sp. phDV1 was cultured M9 minimal at 32 °C and 200 rpm for 72 h. Phenol served as the sole carbon source in the medium at varying concentrations (600 mg/L and 800 mg/L). Growth was monitored by measuring optical density at 600 nm using a UV2700 UV-Vis spectrophotometer (Shimadzu, Kyoto, Japan). Cells were harvested by centrifugation at 6000× *g* for 10 min at 4 °C at OD_600_ = 1.8–2. The pellet underwent two washes with 50 mM Na_2_HPO_4_/NaH_2_PO_4_, pH 7.2, and was subsequently stored at −20 °C. The cultivations were performed in triplicate, ensuring accurate and reliable results.

### 2.2. Phenol Consumption Measurement

Phenol consumption was spectrophotometrically monitored at 277 nm using the Thermo Scientific™ Multiskan Sky Microplate Spectrophotometer (Thermo Fisher Scientific, Waltham, MA, USA).

### 2.3. Nile Red Staining

To prepare the sample, 1–2 mL of cells were centrifuged at 13,000× *g* for 60 s and resuspended in 50 μL of growth medium. In the reaction tube, 1 μL of Nile Red solution [250 μg/mL in dimethyl sulfoxide (DMSO)] was added to 3 μL of cells. Agarose pads were prepared by pipetting 30 μL of hot (60 °C) 1% (*w*/*v*) agarose solution onto a slide. Immediately, 4 μL of the stained cell suspension was added to the agarose pad. After allowing it to dry for a few seconds, a coverslip was placed on top of the agarose. The cells were observed using an oil-immersed lens under a Nikon ECLIPSE E800 microscope (Nikon Instruments Inc., Melville, NY, USA), with an excitation wavelength of 562/40 nm and an emission wavelength of 594 nm.

### 2.4. P(3HB) Quantification by HPLC

The quantification of PHB was determined by measuring the absorbance of crotonic acid at 215 nm using HPLC. The pellet was harvested from 20 mL of cell culture by centrifugation (15,317× *g*, 10 min, 4 °C) and washed twice with equal volumes of acetone and ethanol. The conversion of P(3HB) to crotonic acid was achieved by digesting the pellet in 1 mL of concentrated sulfuric acid (Merck, Darmstadt, Germany) for 30 min at 105 °C. Following digestion, the samples were diluted with nanopure H_2_O in a 1:5 volume ratio and filtered using 0.22 μm filters. Subsequently, the Agilent 1260 Infinity II LC System (Agilent, Santa Clara, CA, USA) was used to analyze the filtered samples. The samples were loaded onto the reversed-phase column InfinityLab Poroshell 120 EC-C18 (4 μm pore size, 4.6 × 150 mm, Agilent, Santa Clara, CA, USA) and eluted with a solution of 0.1 M 85% phosphoric acid (Honeywell, NC, USA) and 15% (*v*/*v*) acetonitrile (Fisher Scientific, Portsmouth, NH, USA) at a flow rate of 0.5 mL/min and a temperature of 30 °C. Crotonic acid in the samples was detected by a diode array detector at 215 nm and quantified based on a standard curve.

### 2.5. Isolation of P(3HB) Granules

Cells were disrupted using an Ultrasonicator Processor UP200s (Hielscher, Bonn, Germany) at 30% amplitude and one cycle (10 times, 15 s at 45 s intervals), ensuring that the temperature remained below 10 °C. Next, approximately 1.5 mL of the broken cell suspension was layered on a discontinuous sucrose gradient consisting of 3 mL of 2.0, 1.67, and 1.33 M sucrose in 0.1 M Tris-HCl, pH 7.5. Following ultracentrifugation at 210,000× *g* for 3 h at 4 °C, the white layers at the interface of 1.33 M and 1.67 sucrose corresponding to the PHB granules were collected. The granules were then washed with 0.1 M Tris-HCl pH 7.5 to remove the sucrose. After washing, the granules were resuspended in 0.1 M Tris-HCl, pH 7.5, and stored at −20 °C for further analysis.

### 2.6. Sample Preparation for the LC-MS/MS Analysis

Samples of the isolated carbonosomes were treated according to the S-Trap protocol [17]. From each carbonosome sample, 100 μg of total protein was homogenized in 100 µL lysis buffer (5% SDS, 50 mM triethylammonium bicarbonate (TEAB), pH = 7.55). The solution was incubated with 20 mM tris(2-carboxyethyl)phosphine (TCEP) for 30 min at 37 °C, followed by incubation with 40 mM iodoacetamide (IAA) for 20 min at room temperature in the dark. After the addition of H_3_PO_4_ (final concentration about 1.2%), 700 µL binding buffer (in a ratio of 1:7 v:v of SDS lysis buffer to S-TRAP binding buffer) was added. The solution was loaded onto spin columns and was centrifugated in a benchtop centrifuge for 30 s at 4000× *g*. The trapped protein was washed three times by 400 µL S-TRAP binding buffer. A total of 125 µL of 50 mM TEAB buffer, pH = 8, containing trypsin was added on the top of the column and was incubated for 1 h at room temperature in the dark. The peptides were eluted with 50 mM TEAB and 0.2% formic acid and subsequently with 50% acetonitrile and 0.2% formic acid. To the pooled eluates, 500 µL 0.1% trifluoroacetic acid (TFA) was added, and the solution was desalted with C18 SPE (Isolute C18) and dried in a speedvac. The pellet was resuspended in 40 µL 0.1% formic acid for mass spectrometry (MS).

### 2.7. MS/MS Protein Identification and Quantification

For the LC-MS/MS injection, the dried peptide fractions were reconstituted in a solution of 5% acetonitrile and 0.1% formic acid, then loaded onto a nano-HPLC (Dionex U3000 RSLCnano) using reverse-phase columns (trapping column: 3 mm particle size, C18, 20 mm length; analytical column: <2 mm particle size, C18, 50 cm length, PepMap, Dionex/Thermo Fisher Scientific). Peptides were eluted using gradients of water (buffer A: water with 5% [*v*/*v*] acetonitrile and 0.1% formic acid) and acetonitrile (buffer B: 20% [*v*/*v*] water, 80% [*v*/*v*] acetonitrile, and 0.1% formic acid). All solvents used were of LC-MS/MS grade and sourced from Fluka. The gradients were increased from 4% to 48% B over 178 min at a flow rate of 300 nL/min. Peptides eluting from the column were ionized in real time using a Thermo nano Flex ESI source and analyzed with a Thermo Orbitrap Eclipse Tribrid Mass Spectrometer. Mass spectra were collected over a range of 350–1600 *m*/*z*, and sequence information was obtained through computer-controlled, data-dependent automated switching to MS/MS mode, utilizing collision energies tailored to the mass and charge state of the candidate ions (TOP15, “FT-IT” mode, MS resolution 120 k, MS2 injection time: 50 ms). Each condition was analyzed in three biological replicates of LC-MS/MS runs.

### 2.8. Data Analysis

The raw files were loaded into MaxQuant (version 2.4.13.0) and analyzed with the Andromeda peptide search engine [18,19]. In brief, spectra were matched to the *Pseudomonas* sp. phDV1 database (UP000258809). The precursor mass tolerance was established at 20 ppm for the initial analysis and 4.5 ppm for the comprehensive search. The fragment mass tolerance was set to 0.5 Da. The search parameters included strict trypsin specificity, permitting up to two missed cleavages. Methionine oxidation (+15.995) and N-terminal acetylation of proteins (+42.011) were designated as variable modifications, while carboxyamidomethylation (+57.021) was fixed. The false discovery rate (FDR) for proteins and peptides was set at 0.01, and only proteins with two peptides were considered for further analysis. Label-free quantification was conducted using MaxQuant, as outlined in [19]. In MaxQuant, the “match between runs” feature was utilized. Protein abundances were determined using normalized spectral protein intensities (label-free quantitation, LFQ). Statistical analysis was carried out using Perseus (version 2.0.3.1). After the removal of contaminants, the quintile-normalized LFQ data were submitted to a *t*-test and permutation-based FDR (FDR = 0.01, S_0_ = 0.1). For further visualization and analysis, R programming language was employed, specifically the libraries “tidyverse”, “psych”, and “ggrepel”.

## 3. Results and Discussion

### 3.1. Utilization of Phenol by Pseudomonas sp. phDV1

**Figure 1:**
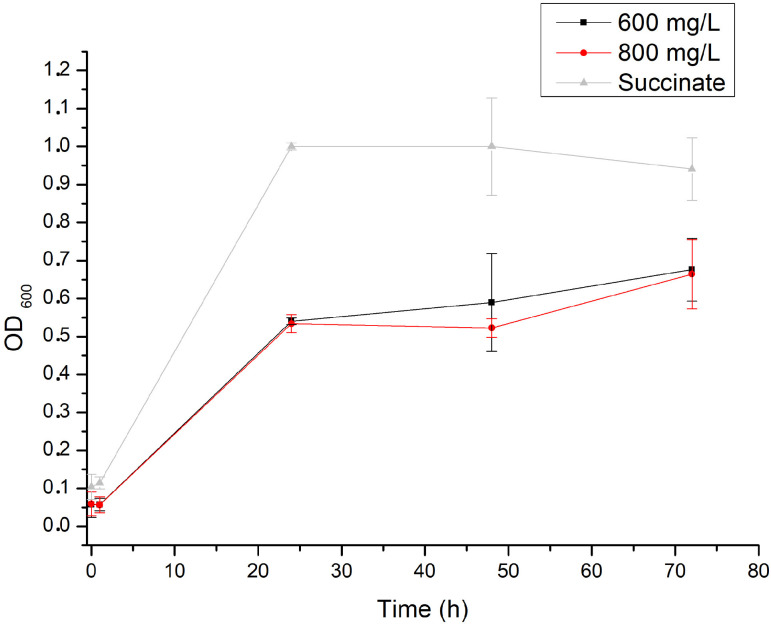
Growth curves of *Pseudomonas* sp. phDV1 in M9 minimal medium in the presence of 10 mm succinate (gray triangles), 6.4 mM phenol (black squares), and 8.5 mM phenol (red circles).

To investigate the effects of different concentrations of phenol on bacterial growth, *Pseudomonas* sp. phDV1 was cultured in M9 minimal medium supplemented with 600 or 800 mg/L phenol as the sole carbon source, respectively. Cell growth was monitored by measuring the OD at 600 nm for 72 h (Figure 1). The results showed that *Pseudomonas* sp. phDV1 was able to degrade phenol at concentrations up to 800 mg/L, which is the highest concentration compared to other studies [20]. As shown in Figure 1, the growth rate of the strain was similar at both concentrations of phenol. Furthermore, the maximum cell density was observed at the stationary phase in all phenol concentrations.

### 3.2. Accumulation of PHB Granules

**Figure 2:**
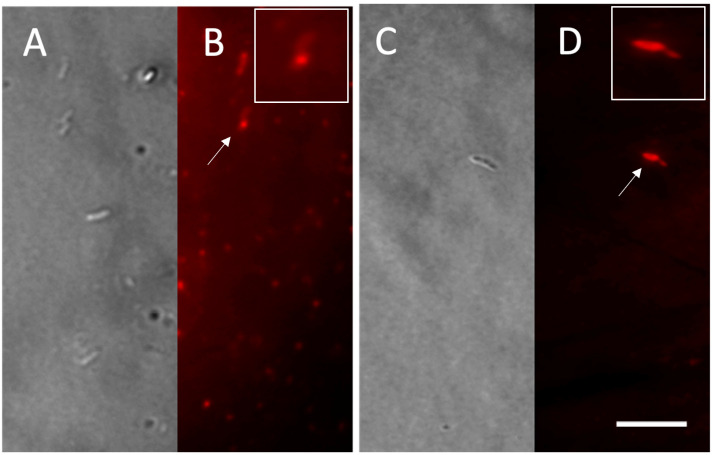
Accumulation of PHB in *Pseudomonas* sp. phDV1 strain. (**A**,**C**) Optical microscopy of *Pseudomonas* sp. phDV1 grown in 6.4 mM and 8.5 mM phenol, respectively. (**B**,**D**) *Pseudomonas* sp. phDV1 strain expressed fluorescence when stained with Nile Red grown in 6.4 mM and 8.5 mM phenol, respectively. PHB production is seen as red fluorescence. Arrows indicate the cell in the insert. Scale bar represents 5 μm.

The *Pseudomonas* sp. phDV1 strain can utilize monocyclic aromatic compounds as a carbon source, such as wine industry waste [21]. The ability of *Pseudomonas* sp. phDV1 to produce PHB has been confirmed previously [6,20]. In this study, we investigated the ability of this bacterium to utilize phenol at high concentrations to produce PHB. The PHB accumulates as granular inclusions in the cell cytoplasm, which can be visualized by different microscopic methods [6,20,21]. The light microscopy findings indicate that *Pseudomonas* sp. phDV1 strain was able to produce PHB after cultivation in the different phenol concentrations (Figure 2).

**Figure 3:**
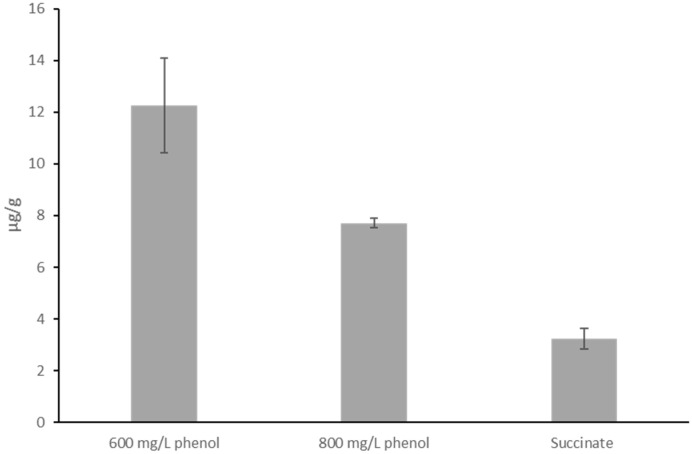
Effects of different phenol concentrations as carbon sources on PHB production from *Pseudomonas* sp. phDV1 in M9 medium containing 6.4 mM, 8.5 mM phenol and 10 mM succinate. The y-axis represents the μg of crotonate per g of dry cells that were in each sample.

To confirm these qualitative findings, the actual concentration of PHB produced by the *Pseudomonas* sp. phDV1 strain grown in 600 and 800 mg/L phenol, respectively, was quantified by HPLC after 72 h of growth. The highest PHB yields were obtained when 600 mg/L phenol was used as the sole carbon source (Figure 3). Proteomic studies have shown that the carbon source affects the abundance of enzymes that are important for the degradation of phenols and cresols [6]. Recent comparative proteomic analyses show that the presence of monocyclic aromatic compounds differentially affects the expression of granule-associated proteins and the proteins involved in PHB synthesis [6]. This observation suggests that PHB production is not correlated with the amount of the carbon source but probably with the abundance of the proteins involved in phenolic degradation and PHB synthesis.

### 3.3. Isolation and Proteomic Analysis of PHB Granules

To study the protein composition of the PHB granules accumulated in the cells, we decided to isolate native carbonosomes and identify the protein present by proteomic analysis. The fraction collected at the interface between 1.33 and 1.67 M sucrose represents the carbonosomes [22]. The presence of P(3HB) in the carbonosomes was confirmed by ^1^H-NMR analysis. The ^1^H NMR spectra of the isolated PHB carbonosomes in CDCl_3_ solution showed a signal at δ 5.26, at δ 2.61, and at δ 1.47, with integral ratios of 1:2:3, respectively (Figure S1). These spectral data (chemical shift and scalar couplings) are identical with those reported for PHB isolated from *Pseudomonas* sp. phDV1 [20,21]. To overcome signals arising from impurities in the sample, we recorded a 2D homonuclear ^1^H-^1^H gCOSY NMR experiment of the isolated PHA material, shown in Figure S1, which shows correlations between neighboring protons linked by scalar *J* couplings. In the gCOSY 2D NMR spectrum, the methine proton (2) is clearly reported for the methyl protons (1) of PHB [20].

**Figure 4:**
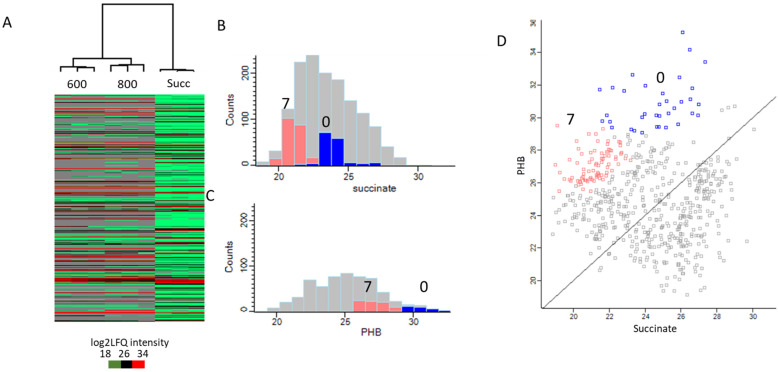
(**A**) Heatmap showing the hierarchical clustering of all samples. Succ represents succinate whole-proteome samples, with 600 and 800 representing the isolated carbonosome samples from phenol at 6.4 mM and 8.5 mM, respectively. (**B**) Histograms of the mean distribution of the log2LFQ intensities of the succinate samples. The red represents the distribution of cluster 7 and blue cluster 0, the two clusters of interest. (**C**) Histograms of the mean distribution of the log2LFQ intensities of the six P(3HB) samples. (**D**) Scatter plot of the mean log2LFQ intensities comparing the P(3HB) samples against the negative control (succinate) whole-proteome samples.

The isolated carbonosomes after cultivation in 600 mg/L and 800 mg/L phenol in three biological replicates were subjected to proteome-wide label-free quantification. Using a modified S-Trap protocol, 873 proteins were identified using the dataset of the *Pseudomonas* sp. phDV1 strain (Figure S2). Hierarchical clustering analysis showed a good correlation between the three biological replicates (Figure 4A), which was also confirmed by a multi-scatter plot and Pearson’s correlation (Figure S3). Compared to comparative proteomic studies of carbonosome fractions from *Ralstonia eutropha* H16, *Caulobacter crescentus, Pseudomonas putida*, and *Pseudomonas putida* ΚΤ2440 show a similar number of identified proteins [14,23,24,25,26,27,28].

Carbonosome-associated proteins are generally predicted to be soluble, or in some cases, to have a hydrophobic region predicted to be a transmembrane helix. To identify proteins that are unique to the carbonosome granule, we compared the proteins found in the granules to the total proteome of the strain grown in succinate [6]. It has been reported that under these conditions, proteins involved in P(3HB) synthesis are less abundant [6]. Furthermore, the comparative proteomic analyses showed that the presence of monocyclic aromatic compounds affects the abundance of granule-associated proteins, especially phasins [6]. Approximately 1500 proteins were identified in the presence of 10 mM succinate and deposited in the PRIDE database (accession number PXD021237). Considering that the strain had a low abundance of proteins involved in the P(3HB) metabolism in the presence of 10 mM succinate, this was used as negative control to detect the possible carbonosome subproteome [6].

The 883 proteins that were identified in the carbonosome samples and the whole-proteome sample were merged. Then, only proteins that were identified in at least three biological replicates of the same condition were retained in the post-filtered dataset, resulting in 1465 proteins (Table S1). A further 1D enrichment analysis was performed on the 883 identified proteins and significand GOBP were carried out (Figure S4). Specifically, this figure shows a bar plot of how many proteins in a stack were significantly overrepresented in each biological sample. The most enriched terms (meaning that this GOBP stood out in at least five biological samples) were translation, sodium ion transport, phosphorylation, outer membrane assembly of Gram-negative bacteria, fatty acid biosynthesis process, and cytochrome complex assembly.

Finally, K-means clustering was performed using the log2LFQ intensities without any normalization or imputation, so the missing values in the isolated carbonosome samples are considered as undetected proteins. The clustering analysis yielded 10 clusters based on the log2LFQ intensities and two clusters (cluster 0 and 7 in a total of 390 proteins, Supplementary Table S1 and Figure 4B–D), which had high abundance in the isolated carbonosome samples and low abundance in the whole-proteome samples (with succinate as a sole carbon source).

In order to remove possible contaminants, a second round of filtering was performed on the list of possible carbonosome-associated proteins. The criteria were that the proteins had to be identified in all biological replicates of the isolated carbonosome granules in the different phenol concentrations and resulted in a list of 118 proteins (Table S2).

Similar to other studies, we found the known proteins related to P(3HB) synthesis [9,29]. Two synthases were found as class I poly(R)-hydroxyalkanoic acid synthase (phaC/DZC76_00035) and poly(3-hydroxyalkanoate) synthase (DZC76_00075). The presence of more than one phaC gene has been reported for different P(3HB) producers [23,30]. In contrast to other studies, we found only one phasin family protein (DZC76_00080) on the PHB granules [29]. The repressor phaR protein is involved in the regulation of PHB synthesis through its interaction with carbonosome granules [14,21]. Although we identified the phaR protein in the PHB proteome, phaR was not present in these two clusters. Another mentioned carbonosome-associated protein, PHB depolymerase (phaZ), was not identified in any of the samples, suggesting that its location may not be on the granule, contrary to previous studies [24,25]. Although high-resolution LCMS technology was used, it was not possible to identify the phaZ protein, which may be due to the use of different isolation protocols. It is also known that the sizes of granules depend on the growth time of bacteria [26]. Isolation at different growth times of the strains may also have an effect on their protein composition.

Recent studies of P(3HB)-associated proteins have also revealed the presence of other proteins with no obvious function in P(3HB) metabolism [27,28,31]. Real-time quantitative analysis of P(3HB) accumulation in living single cells showed that carbonosome formation, growth, and distribution affected the cell volume and length, suggesting the interplay of additional proteins [26]. In addition, 16 proteins were related with metal ion binding, 10 with electron transfer activity, three with transmembrane signaling receptor activity/signal transduction, three with chemotaxis, three with oxidoreductase activity using NAD(P)H, quinone, or a similar compound as the acceptor, and three with sodium ion transport. Based on the literature and in agreement with our results, two outer membrane proteins, namely OmpA and OmpW, were identified in the isolated carbonosome samples [32]. Furthermore, several lipoproteins and the LPS-associated proteins LptE and D were identified in the carbonosome granules, suggesting a possible association with the carbonosomes. The association with carbonosome was confirmed by fluorescence microscopy and localization experiments [25]. Although outer membrane proteins have been identified in other *Pseudomonas* strains, it is possible that these proteins bind to the carbonosome during cell lysis [24,25].

**Figure 5:**
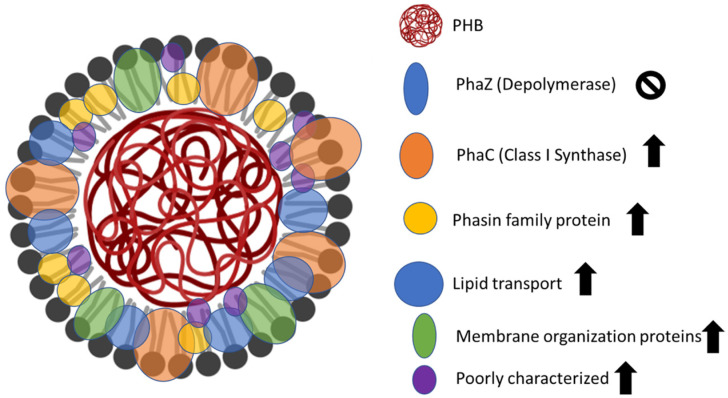
Model illustrating the proteins and processes associated with the carbonosome affected by the growth of the *Pseudomonas* sp. phDV1 strain in phenol based on the fold changes in the proteins.

Furthermore, previous studies have shown DNA-associated proteins that we identified, but which are not included in the 118 proteins that we propose to be PHB-related. Finally, in agreement with the literature, our results also indicated the presence of proteins related to energy metabolism and electron transport [33]. Figure 5 shows the changes in protein abundance and the affected processes on the isolated carbonosomes.

## 4. Conclusions

*Pseudomonas* sp. phDV1 has been shown to be able to produce P(3HB) using phenol as a carbon source in concentrations of 600 mg/L and 800 mg/L. The lowest P(3HB) production was obtained at 800 mg/L, which can be an effect of the toxicity of the high phenol concentration. In addition, finding the optimal growth temperature is crucial to maximizing P(3HB) yield. Most P(3HB)-producing bacteria perform best at 30–35 °C, with variations leading to reduced production efficiency. Further experiments to understand the effects of temperature will help to optimize biological processes for large-scale P(3HB) production. Carbonosomes have been isolated from cell extracts, and label-free comparative proteomic analysis allows for the identification and quantitation of carbonosome-associated proteins. The proteomic results of this study suggest the presence of proteins that are not involved in P(3HB) metabolism on native carbonosomes. Further research is therefore required to elucidate the structure and function of carbonosomes.

## Data Availability

The original contributions presented in the study are included in the article/Supplementary Material, further inquiries can be directed to the corresponding author.

## Supplementary Materials

The following supporting information can be downloaded at: https://www.mdpi.com/article/10.3390/microorganisms13020369/s1, Figure S1: (A) 1D NMR H^1^ of the isolated carbonosomes in 600 mg/L (red) and 800 mg/L phenol as a carbon source. (B) gCOSY NMR H^1^-H^1^ for validation; Figure S2: Bar chart showing the total number of the identified proteins under the two conditions for the isolated PHB samples and the dataset overall. Figure S3: Multi-scatter plot showing the distributions and the Pearson’s correlation of the samples. Figure S4: 1D enrichment analysis (*p*-value 0.05) showing the overrepresented biological processes in the identified proteins. Table S1: Identified proteins in *Pseudomonas* sp. phDV1 grown in succinate and of isolated carbonosome after growth in in 600 mg/L and 800 mg/L phenol as a carbon source. Table S2: Identified proteins with high abundance in the isolated carbonosomes.
