# Supplementary material for: Isolation and Characterization of Carbonosomes from Pseudomonas sp. phDV1 Grown Using Phenol as Carbon Source
Source: Microorganisms. 2025 Feb 8;13(2):369. doi: 10.3390/microorganisms13020369 (PMC11858518; doi:10.3390/microorganisms13020369)
Supplement: Supplementary file 1 [file microorganisms-13-00369-s001.zip › Geladas Microorgansm SM revision.pdf]

Supplementary Material

Isolation and Characterization of Carbonosomes from *Pseudomonas* sp. phDV1 Grown Using Phenol as Carbon Source

Ermis Dionysios Geladas[1¶], Alexandros Lyratzakis[1¶], Athina Drakonaki[1#], Georgios Gkikas[1] and Georgios Tsiotis[1*]

[1] Department of Chemistry, University of Crete, 70013 Voutes, Greece

¶ Equal contribution

# Present address: Center for Soft Nanoscience and Institute of Medical Physics and Biophysics, Busso-Peus Str. 10, 48149 Münster, Germany

***** Correspondence: tsiotis@uoc.gr; Tel.: +30-281-054-5006

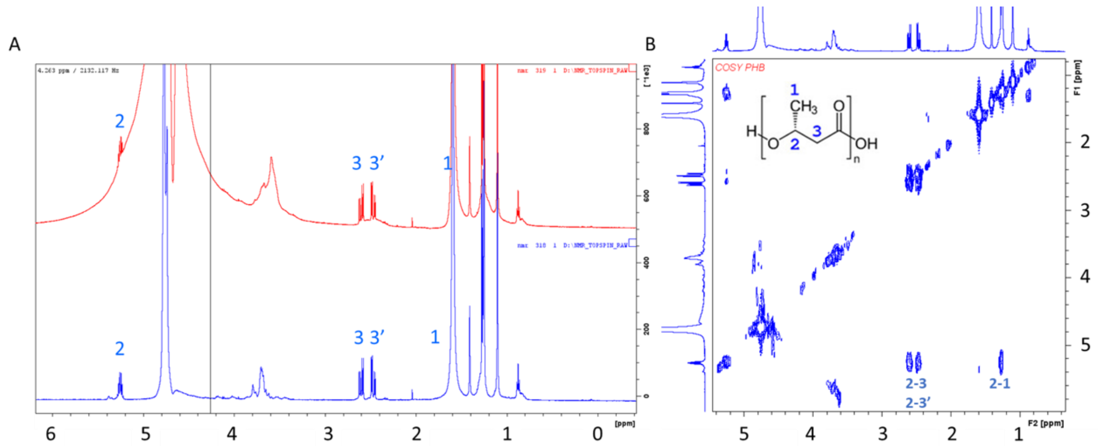

Figure S1. A) 1D NMR H^1 of the isolated carbonosomes in 600mg/L (red) and 800 mg/L phenol as carbon source. B) gCOSY NMR H^1- H^1 for validation.

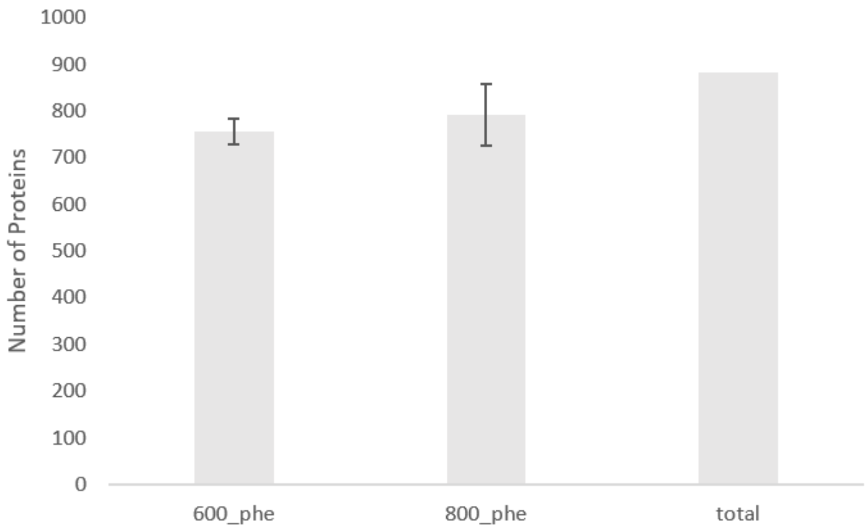

Figure S2. Bar chart showing the total number of the identified proteins in the two conditions for the isolated PHB samples and the dataset overall.

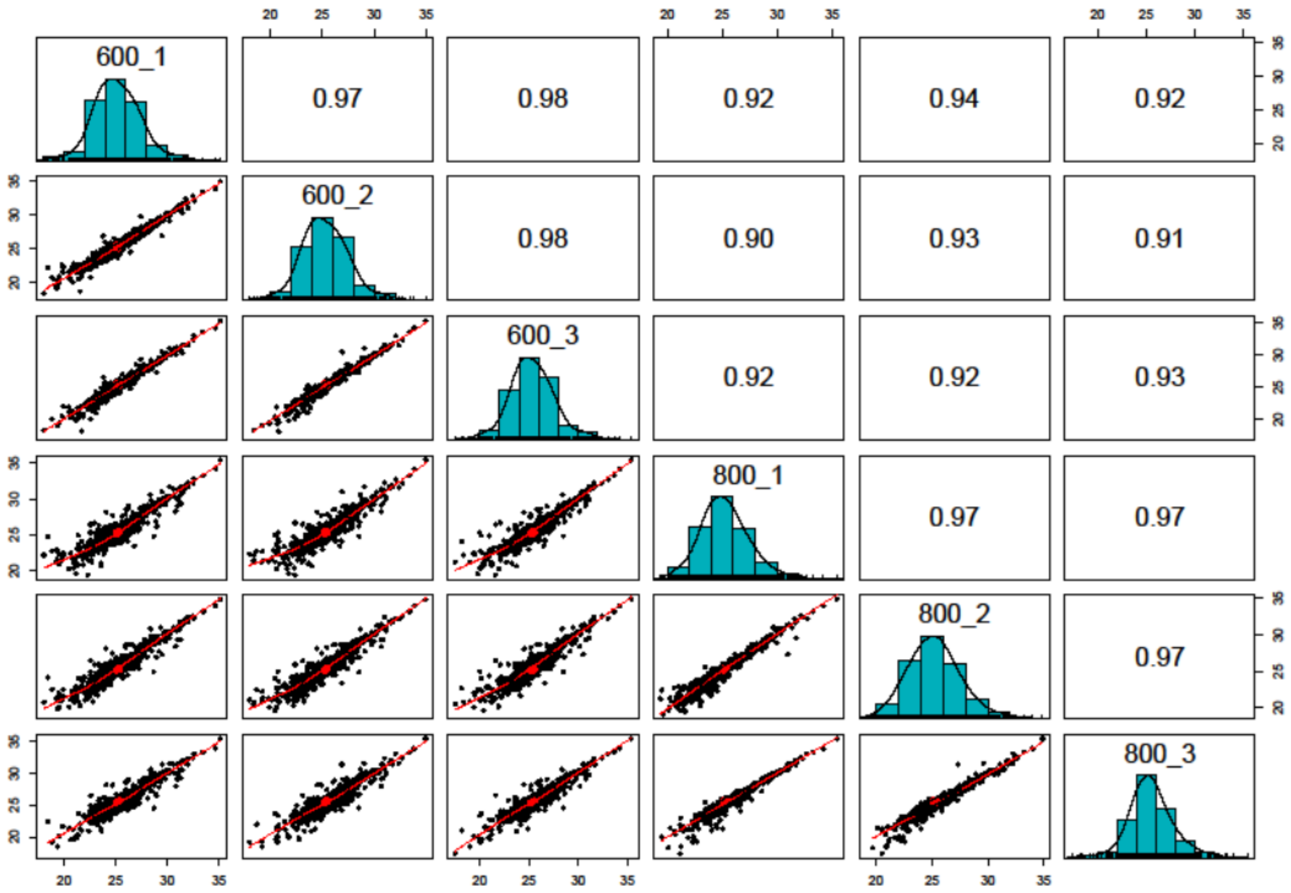

Figure S3. Multi scatter plot showing the distributions and the Pearson's correlation of the samples.

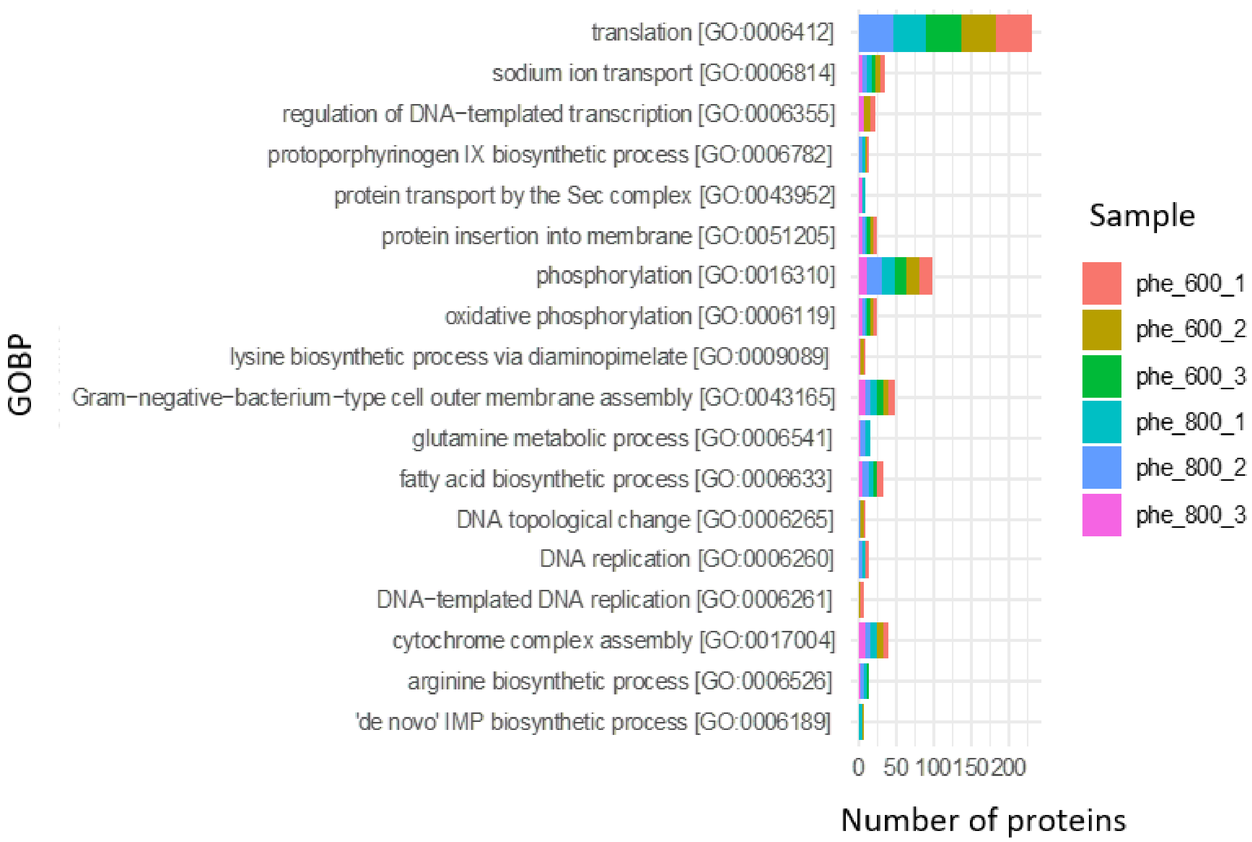

Figure S4. 1D enrichment analysis (p-value 0.05) showing the overrepresented biological processes in the identified proteins.
